# Residents working with Médecins Sans Frontières: training and pilot evaluation

**DOI:** 10.1186/s13049-020-00778-x

**Published:** 2020-08-25

**Authors:** Alba Ripoll-Gallardo, Luca Ragazzoni, Ettore Mazzanti, Grazia Meneghetti, Jeffrey Michael Franc, Alessandro Costa, Francesco della Corte

**Affiliations:** 1 CRIMEDIM, Research Center in Emergency and Disaster Medicine, Università del Piemonte Orientale, Via Lanino 1, PC 28100 Novara, Italy; 2 Médecins Sans Frontières-Italy, Rome, Italy; 3 Department of Emergency Medicine, University of Alberta, Edmonton, AB Canada

**Keywords:** Humanitarian aid, Education, Residents, Evaluation, Low-resource environments, Simulation, Training, E-learning

## Abstract

**Background:**

Well-prepared humanitarian workers are now more necessary than ever. Essential to the preparation process are: clearly defined learning objectives, curricula tailored to the nuances of humanitarian settings, simulation-based training, and evaluation. This manuscript describes a training program designed to prepare medical residents for their first field deployment with Médecins Sans Frontières and presents the results of a pilot assessment of its effectiveness.

**Methods:**

The training was jointly developed by the Research Center in Emergency and Disaster Medicine- CRIMEDIM of the Università del Piemonte Orientale, Novara, Italy, and the humanitarian aid organization Médecins Sans Frontières- Italy (MSF-Italy); the following topics were covered: disaster medicine, public health, safety and security, infectious diseases, psychological support, communication, humanitarian law, leadership, and job-specific skills. It used a blended-learning approach consisting of a 3-month distance learning module; 1-week instructor-led coaching; and a field placement with MSF. We assessed its effectiveness using the first three levels of Kirkpatrick’s training evaluation model.

**Results:**

Eight residents took part in the evaluation. Four were residents in emergency medicine, 3 in anesthesia, and 1 in pediatrics; 3 of them were female and the median age was 31 years. Two residents were deployed in Pakistan, 1 in Afghanistan, 1 in the Democratic Republic of Congo, 1 in Iraq, 2 in Haiti and 1 on board of the MSF Mediterranean search & rescue ship. Mean deployment time was 3 months. The average median score for the overall course was 5 (excellent). There was a significant improvement in post-test multiple choice scores (*p* = 0.001) and in residents’ overall performance scores (*P* = 0.000001).

**Conclusion:**

Residents were highly satisfied with the training program and their knowledge and skills improved as a result of participation.

**Trial registration:**

This study was approved by the Institutional Ethics Committee (date 24-02-2016, study code UPO.2015.4.10).

## Introduction

Widespread global health inequalities and the resulting shortage of humanitarian health workers have led to an increased presence of young doctors in disaster and humanitarian crises [1]. However, although well-prepared humanitarian workers are more necessary than ever [2], traditional medical education struggles to meet the demands posed by globalization and the dramatic escalation of violence [3].

Training objectives for physicians working with Médecins Sans Frontières (MSF) differ from the set of skills acquired in medical schools and residency programs. These professionals are in fact confronted with unique challenges and ethical dilemmas [3] including, but not limited to, different spectra of diseases, limited resources, cultural diversity, and social disruption. For these reasons, training objectives should be set far beyond individual job-specific skills and incorporate a set of core medical and non-medical competencies that all humanitarian workers must possess [4]. Essential to the preparation process before first deployment are therefore clearly defined learning objectives, curricula tailored to the nuances of humanitarian settings, state-of-the-art teaching tools, including high fidelity simulation, and assessment to determine competency [5].

Despite the need for curricula built on a testable package of knowledge and skills, evaluating the effectiveness of training programs is generally not an essential component of preparedness for humanitarian health workers [6], or at best is limited to measuring satisfaction or knowledge. Assessing whether students improve their ability to handle complex situations is of paramount importance to guarantee the best outcomes for vulnerable populations; for this reason, this manuscript describes a training program designed to prepare medical residents for their first deployment with Médecins Sans Frontières and presents the results of a pilot assessment of its effectiveness.

## Materials and methods

### Program development

#### Target Population Survey

In 2012, we conducted a nationwide survey to investigate the interest of young doctors in humanitarian assistance [7]. The survey included all residents in anesthesia and intensive care in Italy. Out of 924 respondents (RR 67.8%), 691 (74.7%) would have liked to make a major contribution to international humanitarian health care during their residencies and 897 (97%) would welcome specific training prior to deployment. Building on these results, we assumed that our training program would be of interest to doctors training in this discipline.

#### Institutions involved

In 2013, the Research Center in Emergency and Disaster Medicine-CRIMEDIM of the Università del Piemonte Orientale (Novara, Italy) in collaboration with Médecins Sans Frontières Italy (MSF-Italy) developed Humanitarian Medic [8], a competency-based training program delivered annually to senior residents in anesthesia and critical care before their first deployment to MSF field missions. In 2015, the course was expanded to residents in emergency medicine and pediatrics.

#### Candidate selection

The number of participants cannot not exceed 10 per iteration; the course includes national and international senior residents (IV-V year) in anesthesia & critical care, emergency medicine, and pediatrics. Selection criteria coincide with the minimum MSF standard requirements for humanitarian workers, namely:

- At least B1 level proficiency in French and English (the languages of health care in the United Nations) according to the European Language Framework;
- Willingness to participate in international humanitarian field projects, including in armed conflict areas or following natural or man-made disasters;
- Flexibility and a positive attitude to working in multicultural contexts.

Prior participation in international cooperation projects or humanitarian emergency response programs is considered an asset but is not mandatory for application.

Candidate selection is carried out in a biphasic fashion by a recruitment commission composed of two CRIMEDIM investigators and two recruiters from MSF-Italy human resources department. Participants are first screened based on their curriculum vitae, self-assessed theoretical and practical skills and the results obtained in an on-line French and English language test. Subsequently, the best candidates are selected for interview.

#### Curriculum

Educational needs were established on the basis of an expert opinion survey [9], round tables with CRIMEDIM and MSF field experts, and a literature review of published competency sets for humanitarian workers [10]. Since our training program targeted health workers but was meant to be extendable in the future to other sectors operating under the umbrella of humanitarian aid, four manuscripts were selected on the basis of their cross-sectorial approach [6] and definition of discipline-specific competencies relevant to our audience [11–13]. These competency sets served as a foundational basis for the course curriculum and were translated into 10 cross-sectorial and 1 profession-specific competency domain for each specialty [Table 1]. Learning objectives were phrased according to Bloom’s Taxonomy. For each objective, a series of measurable performance goals was further developed. Curricula, learning and performance objectives were reviewed and validated by consensus between CRIMEDIM and MSF-Italy working groups.

**Table 1:** Competency domains, general learning objectives and some performance objectives at the basis of the training curriculum

Competency domain	General learning objectives	Examples of performance objectives
**1. Disaster medicine**	• Understand the definition and different phases of disasters. • Define the nature of injury or illness in relation to different types of disasters. • Describe objectives and features of disaster medicine. • Understand the international disaster response mechanism with involved bodies and organizations.	• List the four phases of disaster management • Name the office of the United Nations responsible for the international coordination in case of disaster or humanitarian emergency
**2. Incident Management System (IMS)**	• Describe the general principles and different phases of the IMS. • Demonstrate ability to work within an IMS. • Describe the concept and different methods of Mass Casualty Triage. • Define the concept of surge capacity and its role in unforeseen emergencies and disasters.	• Correctly carry out the initial reporting from a simulated disaster site using the METHANE (Major accident, Exact location, Type of accident, Hazards, Access, Number of victims, Emergency services) method. • Assign simulated victims with the correct priority code according to the Simple Triage And Rapid Treatment (START) triage.
**3. Communication**	• Recognize a disaster in progress, assess and report the situation. • Define and apply the principles of successful communication with local and expatriate staff, within and among organizations and with the media during emergencies. • Describe the radio communication procedures and protocols. • Recognize the importance of post-event reports.	• Implement the basic principles of communication in a public release statement with the media regarding the attack of a health facility by one belligerent party. • Write and present a post-event report after a simulated mass casualty event summarizing the facts occurred and the actions taken. • Successfully collaborate with a member of local staff with very limited English speaking skills during the clinical management of a simulated critically -ill patient.
**4. Resource management**	• Manage supplies, drugs and equipment and other resources for an effective response. • Manage, supervise, and appropriately use local staff and expatriate aid workers during emergencies.	• Consider early blood compatibility testing for relatives of patients in an hemorrhagic shock scenario when whole blood is scarce or not available. • Demonstrate competence in the use of outdated equipment (e.g ventilators) to provide safe anesthesia in a low-resource-setting.
**5. Public health**	• Recognize the top priorities for public health interventions during complex emergencies. • Describe indicators used to assess and monitor public health during complex emergencies. • Understand key epidemiological principles and terminology. • Define the minimum levels to be attained in humanitarian interventions regarding the provision of water, sanitation and hygiene. • Define the minimum levels to be attained in humanitarian interventions regarding the provision of food and nutrition. • Identify which infectious diseases can constitute a major threat following a disaster according to the geographical location and the type of emergency occurring.	• Describe the information to be gathered during a Initial Rapid Assessment and elaborate an intervention plan according to the identified public health needs. • Name the minimum quantity of safe drinking water (liters/ person/ day) to be provided in an humanitarian intervention. • List the main anthropometric indices used to assess malnutrition. • Demonstrate knowledge about the age groups to be covered by a measles vaccination campaign
**6. Safety and security**	• Understand the need for a safe and secure approach in humanitarian environments. • Analyze the security environment on the basis of the seven pillars of security. • Apply the preventive measures and/or individual or collective responsibilities adapted to each form of stress. • Identify sources of risk, describe risk scenarios and identify risk mitigation measures.	• Demonstrate successful negotiation skills when approaching a simulated check point. • Demonstrate ability to prevent incidents during road travels (e.g carrying ID card, being able to clearly explane the mission of his/her organization etc). • Identify landmine markings during outdoors exercises
**7. Ethics and international humanitarian law**	• Apply basic principles of medical ethics to disaster situations. • Recognize and react accordingly to the difficulties entailed by humanitarian scenarios where different cultural backgrounds are represented. • Define the concept and understand the origins of International Humanitarian Law • List the main International Human Rights • Describe the role of International Humanitarian Law in in protecting the dignity and rights of the most vulnerable populations during armed conflicts	• Demonstrate tolerance when dealing with local staff and patients with different cultural background (e.g covered with burqa). • Describe the origin of the Geneva Convention
**8. Situational awareness**	• Respond appropriately to an ever-changing environment and stress-induced situations. • Adapt to pressure and change to operate effectively within humanitarian contexts.	• Demonstrate avoiding fixation errors during the management of critically-ll patients in simulated low-resource scenarios. • Demonstrate ability to anticipate likely events in crisis situations (e.g a huge number of victims to come after a single patient presents with acute organophosphorus pesticide poisoning in a war context).
**9. Psychological support**	• Describe the main psychological needs in emergency contexts. • Describe the essential criteria to organize actions in psychological support. • Apply the principles of psychological first aid in emergency situations	• Identify and list the basic principles of Disaster Mental Health. Demonstrate ability to provide the principles of Psychological First Aid • Demonstrate ability to develop good practices to manage personal stress in order to mitigate potential adverse effects of stress
**10. Leadership**	• Understand the definition of leadership and recognize the importance in an emergency context. • Describe the different management styles. • Understand conflict management and modify one’s own management style. • Apply the principles of Non-Violent communication.	• Demonstrate ability to implement a Non-Violent communication when giving a member of the local staff a negative feedback regarding his performance during a recent emergency. • Demonstrate ability to make firm decisions during a critical event: e.g. priority of transport for severely injured patients in an hostile environment.
11. **Clinical considerations in the specific field of Anesthesia, Pediatrics and Emergency Medicine in Low Resource Settings**	• Understand and apply the principles of safe anesthesia, emergency medicine or pediatrics in low-resource settings according to the needs and resource available.	• Demonstrate good knowledge in the use of Halothane, ketamine, suxamethonium and pancuronium • Demonstrate ability to perform a newborn resuscitation in a resource-constrained environment • Promptly recognize and treat signs and symptoms of malaria in high risk areas

#### Delivery method

The course intends to expose participants to a blended-learning experience consisting of 3months of distance, self-directed learning and 1 week of residential instructor-led teaching. E-learning takes place through the Modular Object-Oriented Dynamic Learning Environment (MOODLE) educational software hosted on CRIMEDIM’s servers. The platform works as a content-driven learning model, hosting 11 e-modules and video lectures and offering a suite of tools and online-multiplayer-virtual exercises. The residential phase takes place at the SIMNOVA simulation center in Novara, Italy, and includes class-room sessions, table-top exercises, and group discussions with emphasis on high fidelity and outdoor real-size simulation exercises. Scenarios are designed based on the equipment, drugs and diagnostic tools available in MSF field projects and residents are exposed to the challenges most commonly encountered in daily activities. In clinical management scenarios, actors comply with the dress code of the country where the scenario is based and are also trained to act as typical members of the local staff.

E-learning materials and best performances for simulation exercises are jointly developed based on current international guidelines and the typical resources available in real-life MSF missions. Upon successful completion of both phases, students receive a certificate of completion and are deployed to MSF field projects as local staff supervisors to work as part of the hospital duty roster.

### Evaluation

#### Participants

The first two editions in 2013 and 2014 were not included in the evaluation but were offered on a pilot basis to test the feasibility of the project from an organizational standpoint and refine the course contents according to the feedback provided by students and MSF field supervisors.; only participants to the 2015 course iteration (*n* = 8) were included in the evaluation. All were Italian, four were residents in emergency medicine, 3 in anesthesia, and 1 in pediatrics; 3 of them were female and median age was 31 years old. Two residents were deployed in Pakistan, 1 in Afghanistan, 1 in the Democratic Republic of Congo, 1 in Iraq, 2 in Haiti and 1 on board of the MSF Mediterranean search & rescue ship. Mean deployment time was 3 months.

#### Method

The Kirkpatrick’s evaluation model has recently been used to evaluate training programs for health providers and focuses on the sequential assessment of the following levels [14]:

- Level 1- *Reaction:* measures students’ satisfaction with the program;
- Level 2- *Learning:* measures improvement in knowledge, attitudes and skills;
- Level 3- *Behavior*: measures the transfer of learning to the workplace;
- Level 4- *Results:* measures the objective changes occurred as a result of participation in the training program.

To determine the effectiveness of our course, we tested levels 1 to 3 using a prospective, observational, single-cohort study.

#### Evaluation plan and evaluation tools

**Fig. 1:**
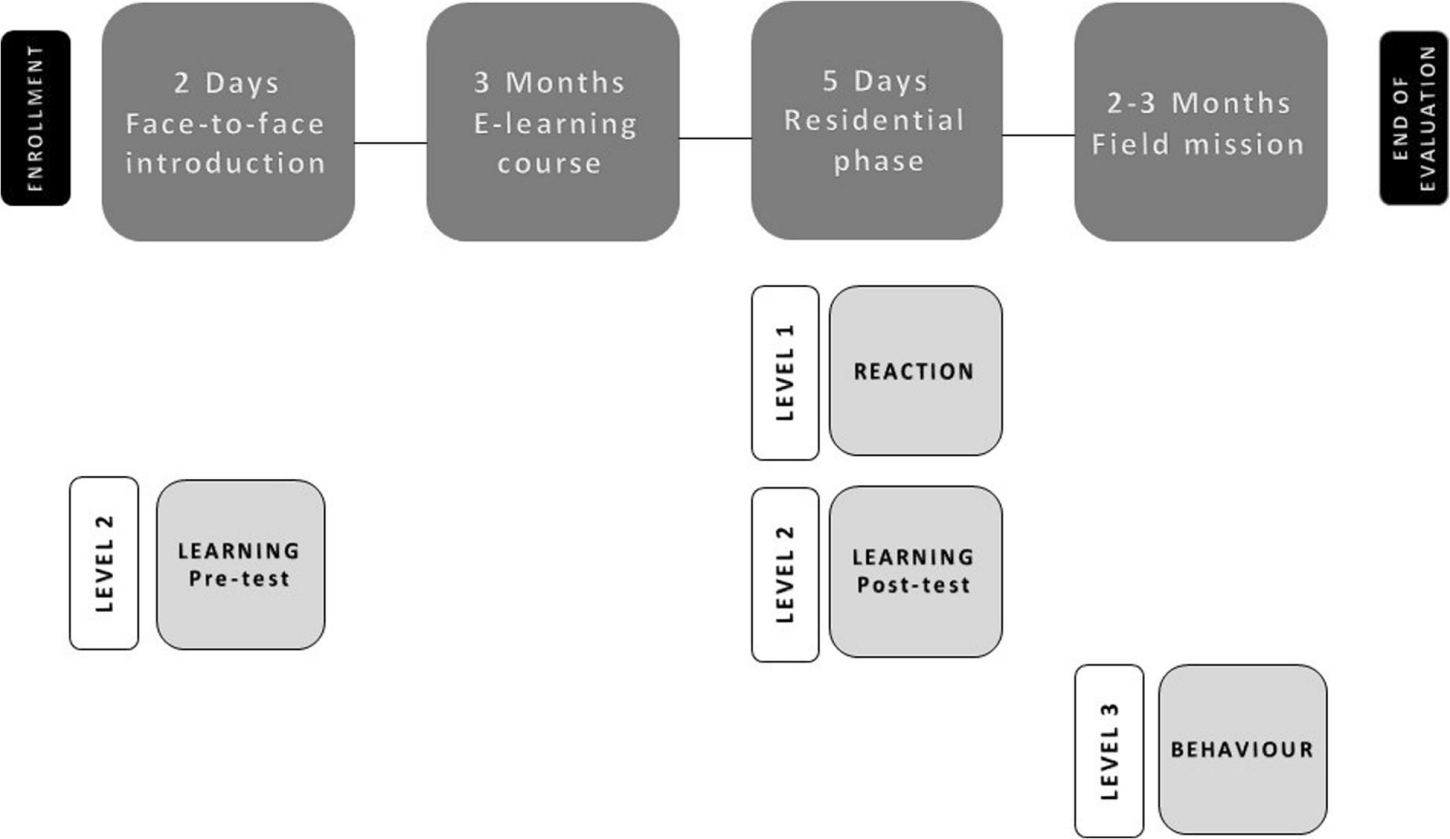
Graphic depiction of the evaluation process. Overall, residents participated in a face- to-face course opening session where Level 2-pre-test was administered. Level 1 and Level 2 (post-test) were assessed at the end of the residential course. Behavior was evaluated at the end of students’ field missions

We designed the evaluation plan according to the recommendations of Kirkpatrick et al. [15] [Fig. 1]:

- We assessed Reaction using a 5-point Likert scale questionnaire with a separate space for commentaries and personal opinions.
- We evaluated the three dimensions of Learning separately in a pre- and post-test [Fig. 1] as follows:
  - Knowledge with a 30-question-multiple-choice test.
  - Attitude with a 12-question-5-point Likert scale questionnaire. In this study, the term “attitude” was defined as the students’ positive or negative predisposition toward the competency domains at the basis of the course.
  - Skills with simulation-based performance tests, in which each student acted as lead physician in the management of a critically-ill patient in a low-resource emergency room. To decrease the potential impact that reiterative exposure to simulation exercises during the residential phase might have had on post-test performance, students attended standardized simulation tutorials and managed a number of simulated cases before entering the pre-test scenario. Pre- and post-test scenarios progressed on a predefined fashion according to the cue system described by Kim et al. [16] We videotaped resident’s performance and passed the material on to an external independent evaluator, who rated all students according to the validated Italian translation (Translated Italian Global Rating Scale- TIGR) [17] of the Ottawa Global Rating Scale (Ottawa GRS) [16]. This scale uses a 7-point semi-anchored design to evaluate residents’ overall performance and their leadership, problem solving, situational awareness, resource utilization, and communication skills.
  - Pre- and post-test were different tests with the same level of difficulty.
- Every resident was followed during the mission by his immediate MSF field supervisor who qualitatively assessed improvements in behavior using the MSF standard evaluation form for first missioners (form available upon request).
- MSF field supervisors were blinded to the students’ completion of the training program before deployment. This was necessary to prevent students’ participation from influencing the judgement of supervisors and ensure that evaluations were carried out as usual for any MSF first missioner.
- All the evaluation tests/forms and case scenarios used to conduct the evaluation are available upon request.

#### Statistical tests and data analysis

The statistical analysis of Level 2 (Learning) was conducted by taking the difference in pre- and post-test scores as measured with a multiple-choice test as the primary objective. The difference in pre-and post-overall performance was defined as the secondary objective. We defined null hypotheses as no difference between the before and after multiple-choice test scores and no difference between the before and after overall performance. Alternative hypotheses were that both multiple choice test scores and overall performance scores would change significantly after completion of the course.

We tested the null hypothesis of the primary objective of *no difference between pre- and post-test scores* against the two-sided alternative hypothesis of significant difference. The null hypothesis of the secondary objective of *no difference between pre- and post-training overall performance scores* was tested against the two-sided alternative of significant difference. Statistical analysis was performed using “R: A language and environment for statistical computing.” (R Development Core Team, Vienna, Austria). Null and alternative hypotheses and the statistical methods were completely specified before data collection. Differences between groups for the primary and secondary objectives were assessed using paired *t*-tests. *P*-values of less than .05 were considered significant for all tests. Two-sided alternative hypotheses were used in all cases.

#### Ethical clearance

To ensure anonymity and confidentiality during the entire evaluation process, we assigned a tracking number to each participant. This number was then reported on answer sheets, evaluation forms and videotape labels. All students signed the informed consent. This study was approved by the institutional Ethics Committee (date 24-02-2016, study code UPO.2015.4.10).

## Results

### Reaction

All residents rated the course as “excellent” and strongly agreed with the statement “I would recommend the course to other doctors in training”. All participants emphasized their satisfaction with both course contents and mode of delivery. In particular, high-fidelity simulation exercises were highly appreciated. Two students expressed the view that the course schedule was too tight and one suggested adding more training in negotiations techniques.

### Learning

**Fig. 2:**
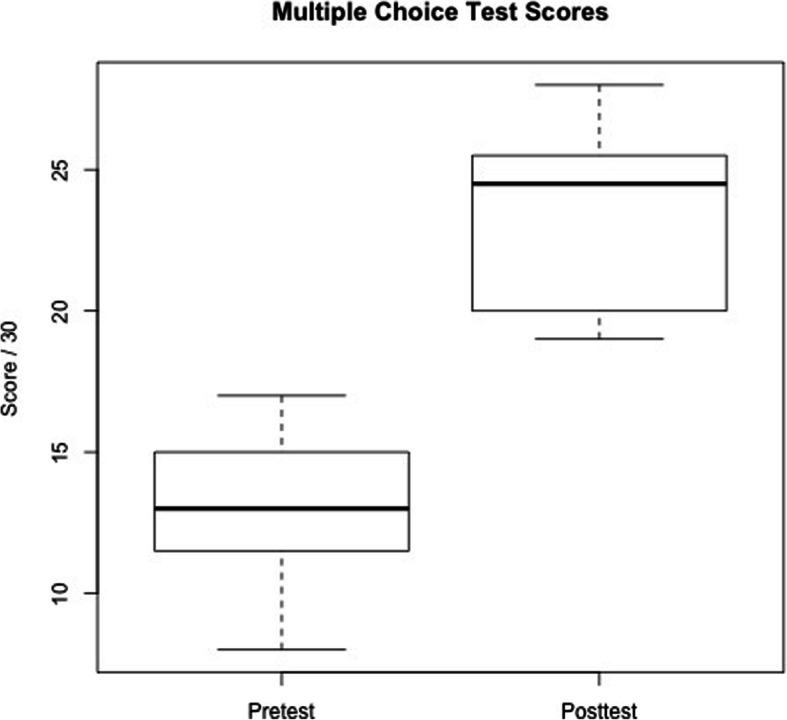
Boxplot representing multiple choice test scores before and after the course

**Fig. 3:**
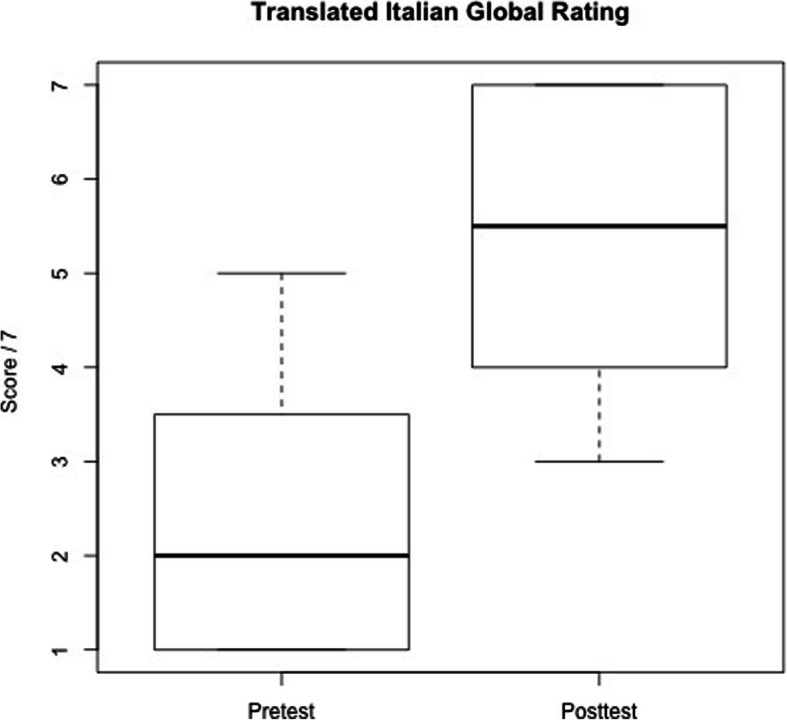
Boxplot representing (TIGR) overall performance test scores before and after the course

There was a significant improvement in post-test multiple-choice scores when compared to pre-test scores (*p* = .001) (mean effect: 10.4/30; 95% CI: 5.7 to 15.0) [Fig. 2] and also a significant improvement in residents’ overall performance scores (*P* = .000001) (mean effect: 3; 95% CI: 2.4 to 3.6) [Fig. 3]. The median score for all other fields also improved after the training course (median differences: Leadership 3.3; Problem solving 2.6; Situational Awareness 3; Resource Utilization 3.5; Communication 2.7). No differences were detected in attitudes scores before and after the course.

### Behavior

For most participants, MFPs highlighted the following strengths: compliance with MSF standards and principles (7), flexibility (6), good team working skills (6) and cross-cultural sensitivity (3). All residents (8) were recommended for future MSF missions [Table 2].

**Table 2:** Summary of evaluations from field supervisors for each candidate

Student	Strong competences	Competences to develop
1	Good analytical thinking, well-organized, high working capacity, good training skills, compliance with MSF standards and principles, flexibility, empathy, good mass casualty management skills, good technical skills, hard work, good team working skills, good reource management skills.	Human resource management Tropical medicine
2	Well-organized, good technical skills, good training skills, hard work, good team working skills, good reource management skills, cross-cultural sensitivity, compliance with MSF standards and principles, good negotiation skills, good comunication skills.	Human resource management
3	Cross-cultural sensitivity, good team working skills, good training skills, good people management skills, good leadership skills, well-organized, good analytical thinking, good problem solving skills, good reource management skills, good decision making skills, responsibility, flexibility, good stress management skills, compliance with MSF standards and principles, implement good strategies to ensure security and safety skills in daily work, good comunication skills, hard worker, worked as a person with more experience in MSF.	Participation in monthy reports
4	Cross-cultural sensitivity, good team working skills, good training skills, compliance with MSF standards and principles, good at motivating local staff, good reource management skills, flexibility, multitasking, deep commitment, hard work.	Self-health care
5	Good mass casualty management skills, compliance with MSF standards and principles, good leadership skills, good communication skills, good team working skills, responsibility, good training skills.	Language skills
6	Good analytical thinking, compliance with MSF standards and principles, good team working skills, good at setting priorities, good clinical skills, good team working skills, good comunication skills, flexibility, multitasking, will to improve organization within the project.	Too ambitious with local staff setting sometimes unrealistic goals
7	Maximum committment to MSF, compliance with MSF standards and principles, cross-cultural sensitivity, very good attitude towards MSF staff, awareness of the project from a global perspective and not only in own area of competence, committment to promote capacity building, good team working skills, good at motivating local staff, flexibility, implemented an operational research project approved by MSF medical coordination unit.	Self-protection during life saving maneuvers
8	Highly adaptable, good skills to work with limited resources, flexibility, responsibility, good at coaching and support of local staff, good reource management skills.	

## Discussion

Expatriate health providers have been observed to be ill-prepared during recent humanitarian emergencies due to lack of experience in international relief and inadequate understanding of the local context [4, 18]. For this reason, the international humanitarian community has been drawing attention to the compelling need for competency-based training curricula based on a standard set of cross-cutting and profession-specific competencies [4, 19]. Since young doctors, born in a new era of highly-specialized medicine, have been increasing their presence in international aid projects, good preparation and performance oversight are paramount to guarantee best practice also in resource-strained settings [9].

To our knowledge, this is the first study that describes the implementation and evaluation of a course based on published cross-sectorial and profession-specific competencies, jointly developed by an academic center and a robust humanitarian organization. Interestingly, while 61% of training programs for humanitarian workers in Europe [19] are defined as “competency-based”, none of them incorporates previously published competency sets. Evaluating the effectiveness of training programs is necessary to ensure credibility and decide whether they should be continued or not. In this regard, the assessment of a course for humanitarian workers using the first three levels of the Kirkpatrick’s evaluation model and including high fidelity simulation represents a real novelty.

Overall, students’ satisfaction (Level 1) with the program was high. In particular, the delivery method and the residential phase were highly appreciated. It is worth noting that, aside from course curriculum and students’ previous academic background, the structure of the program and the educational environment also play a central role in learning [20]. Over the last years, medical education has shifted toward different delivery modes in an attempt to achieve better educational outcomes. The combination of face-to-face lectures and online teaching, defined as blended learning, strengthens the interaction between course participants, lecturers and resources [21] and represents a flexible pedagogical system [22].

Our study showed that students’ competency in simulated humanitarian scenarios increased after course completion (Level 2). The term “competency” is defined as the set of knowledge, attitudes and skills required to accomplish a task. A competency-based training must ensure the acquisition of a theoretical understanding of relevant concepts in the field of humanitarian assistance (e.g., learning the Simple Triage And Rapid Treatment (START) triage algorithm), promote a positive predisposition toward the working methods and actions to be adopted in low-resource settings (e.g., recognizing the importance of applying the START triage), and develop students’ practical skills (e.g., conducting good quality triage in case of a mass casualty event). Yoon et al. [23] used the Kirkpatrick model to evaluate a continuing professional development training for physicians and physician assistants. In their study, a single 5-point Likert scale form filled by trainers and trainees was used to assess learning. However, it is important to highlight that the inclusion of separate tests for knowledge (e.g., multiple choice), attitudes (e.g., Likert scale questionnaire), and skills (e.g., performance test) should be preferred whenever possible [14].

Our results reflect a clear improvement in participants’ knowledge and overall performance in a high-fidelity scenario, while no change in attitude was evident. A plausible explanation may lie in the selection process itself. Since all students were highly motivated and had decided to take part in the course on a voluntary basis, a positive attitude was to be expected.

In a recent study, Schwartz et al. highlighted the prominent role that simulations, and particularly high-fidelity simulations, may play in enhancing residents’ skills in the management of complex cases [24]. Simulated environments are an invaluable setting to teach crisis resource management (CRM) skills [25], which are extremely important in humanitarian contexts. Some challenges commonly encountered in the field (e.g., communication barriers and shortage of resources) can be easily reproduced through simulation, giving students the chance to become acquainted with similar situations, receive feedback and improve their performance with no risks to patients. At the same time, simulated scenarios allow for the evaluation of performance objectives, reflecting how students would use in the field the competencies acquired through training [11]. In their review entitled “Transfer of learning and patient outcome in simulated crisis resource management”, Boet et al. found that CRM simulations improve not only learners behavior in the workplace but also, and more importantly, patient outcomes [26].

According to Kirkpatrick et al., [14] a positive reaction and evidence of improvement in learning do not necessarily lead to desired changes in behavior. The transfer of learning to the workplace is heavily conditioned by the so-called ‘work climates’, and these are clearly established by a supervisor’s reaction to students’ practical application of the competencies acquired. To promote an encouraging work environment, heads of department and supervisors should be informed about the students’ participation in the training program, and preferably be involved in its development [14]. In our case, keeping MSF field evaluators blinded was mandatory to prevent biases; however, all participants received very good feedbacks. The reason for this may be the fact that educational needs were decided and endorsed by a panel of experts that included MSF training staff. This ensured that the practical concepts, organizational principles and techniques taught complied with the organization’s best practice standards.

The collaboration between an academic center and a robust humanitarian organization allowed us to demonstrate the effectiveness of a pre-deployment training course in improving participants’ learning. This may have several promising implications:

1. If properly trained, medical residents with no previous experience in the field could be deployed without compromising the quality of care delivered.
2. In countries where residents are authorized by contract to practice abroad for a certain period of time while maintaining their financial remuneration, non-governmental organizations (NGOs) could fill field gaps more rapidly by deploying well-prepared but inexpensive personnel.
3. From an organizational standpoint, agreements between NGOs and training centers would allow humanitarian staff to benefit from simulation-based training, which is presently the best approximation to real work in emergency and disaster contexts [27].

It is our hope that this collaborative initiative will serve worldwide as a model to bridge the gap between academia and field operations and contribute to the growth and professionalization of the humanitarian health sector.

### Limitations

Despite our efforts to conduct this study thoroughly, a number of limitations should be considered.

The competency sets and skills at the basis of our training curriculum, albeit published and peer-reviewed, were never validated. However, the authors believe that basing the learning objectives of the course on the needs emerged from discussions with different groups of experts would go some way toward remedying this deficiency. Incorporating the input of trainers working for the NGO partner would also be a fair compromise in the absence of a globally recognized competency set for humanitarian workers.

The study dataset was limited to 2015 and the sample size used to test the effectiveness of this course was limited to 8 participants. Since our target population was composed of doctors still at an early stage of their careers, the tight enrollment criteria severely limited the number of eligible applicants for each iteration. Also, the evaluation process was logistically challenging and very resource consuming. All participants had to travel to Novara on purpose for the pre-test and their travel and living expenses had to be covered for 2 days both pre- and post-test. This prevented the evaluation to be repeated in the following editions, which would have positively impacted the sample size. Even though both primary and secondary outcomes improved significantly after the course, a larger study would be useful to confirm the significance of the specific changes found in each field of the TIGR evaluation scale.

This study only assessed the first three levels of the Kirkpatrick model; additionally, for level 3 (Behavior) no pre-test was conducted. Since the course was designed to prepare residents to their first deployment with MSF, exposition to the real workplace environment was only possible upon completion. All participants obtained very good assessments from field supervisors, which may suggest that the training program played a role in the quality of their respective performances in the field.

Level 4 (Results) measures the effect of students’ actions, which considering our target population should have translated into measurement of patients’ outcomes (e.g., decreased mortality). Taking into account the high turnover of doctors in humanitarian contexts and the diversity in the pattern of disease and affluence of patients depending on country, season, and ongoing environmental conditions (armed conflict, natural disaster, etc.), the influence on patients’ outcomes would have been very hard to ascertain.

Finally, this study did not include a control group. According to MSF policy, deploying untrained doctors in the field at this early stage of their careers would have gone against basic principles of best practice.

## Conclusions

Over the last decade, the humanitarian community has stressed the need to improve the quality of response through further investments in training for aid workers. Residents were highly satisfied with our training program and their knowledge and skills in simulated humanitarian environments improved as a result of participation. The implementation of this project shows how academia can successfully partner with humanitarian aid organizations to promote the professionalization of future humanitarian health workers. Further studies should be conducted to assess whether training programs effectively increase the competence of humanitarian workers in the field and if this translates into improvement of patients’ outcomes or further advantages for deploying organizations.

## Data Availability

Complete data and evaluation forms/tests used are available upon request.

## Abbreviations

MSF: Médecins Sans Frontières
CRIMEDIM: Research Center in Emergency and Disaster Medicine of the Università del Piemonte Orientale, Novara, Italy
MOODLE: Modular Object-Oriented Dynamic Learning Environment
TIGR: Translated Italian Global Rating Scale
Ottawa GRS: Ottawa Global Rating Scale
START: Simple Triage And Rapid Treatment
CRM: Crisis resource management
NGOs: Non-governmental organizations
